# A Comparison Between Ultrasound Pachymetry and CASIA2 (Anterior-Segment Optical Coherence Tomography) in the Measurement of Central Corneal Thickness

**DOI:** 10.7759/cureus.39921

**Published:** 2023-06-03

**Authors:** Joe Baxter, Nadeem Atwan

**Affiliations:** 1 Ophthalmology, Sheffield Teaching Hospitals NHS Foundation Trust, Sheffield, GBR

**Keywords:** anterior-segment oct, bland-altman, ocular hypertension, pachymetry, central corneal thickness

## Abstract

Background and objective

Due in part to its effect on intraocular pressure (IOP) measurements, the assessment of central corneal thickness (CCT) is recognized as an essential part of the initial glaucoma assessment. The most widely utilized clinical technique to measure CCT is ultrasound pachymetry (USP). In recent years, many dedicated anterior-segment optical coherence tomography scanners (AS-OCTs) have been developed. Previous studies have compared CCT measurements between USP and various AS-OCTs. This study aimed to assess the degree of agreement between USP and CASIA2 (Tomey Corporation, Nagoya, Japan), a second-generation swept-source AS-OCT developed in Japan.

Methodology

The data on CCT screening measurements of 156 eyes (88 patients) performed over a period of three months, from January to March 2020, on glaucoma patients attending the Royal Hallamshire Hospital (RHH) in Sheffield, UK were collected retrospectively and statistically analyzed.

Results

The average age of the 88 patients included in the study was 66 years (range: 20-86 years). Our findings show that when compared to CASIA2 measurements, USP measurement of the CCT resulted in significantly thicker values (paired t-test: t=23.15,p<2.2 x 10^-16^). The average difference between the two methods was 19.98 ± 10.78 μm. It is hypothesized that this difference may be due in part to inaccurate probe placement during ultrasound probe measurement, resulting in thicker CCT values.

Conclusion

The observed difference may be clinically significant as it could induce clinical discrepancy in terms of perceived glaucoma risk in patients. Therefore, USP and CASIA2 should not be used interchangeably, and clinicians should take into account the significant difference between these methods.

## Introduction

Central corneal thickness (CCT) is a valuable and sensitive measure of corneal health and physiological performance [1]. It can be a crucial factor to consider when monitoring corneal pathologies such as keratoconus, Fuchs’ endothelial dystrophy, and corneal edema [2,3]. CCT is also an essential parameter when evaluating patient suitability for refractive surgery, dry eye therapy, and corneal transplantation [4]. Furthermore, CCT measurement is recognized as an integral part of clinical glaucoma assessments [5-7], as differences in CCT can introduce artifacts to intraocular pressure (IOP) measurement [8]. Utilizing Goldmann applanation tonometry is most accurate when measuring eyes with CCTs of approximately 520 µm; CCTs thicker or thinner than this may lead to over- or underestimations of IOP, respectively [8,9]. Such inaccuracies in IOP readings may lead to patient misclassification in terms of glaucoma risk [10].

Additionally, thin CCT has been suggested as an independent risk factor for glaucoma. The Ocular Hypertensive Treatment Study (OHTS), by controlling for IOP, highlighted thin CCT as a significant risk factor for the development of glaucoma from ocular hypertension (OHT) [11]. The OHTS indicated that OHT patients with CCT below 555 µm had a three-fold higher risk for developing primary open-angle glaucoma (POAG); the study concluded that a thin CCT is the single most important risk factor for the development of glaucoma.

Since the publication of OHTS findings, research interest in the importance of CCT has surged. Before 2002, the number of yearly publications regarding CCT and glaucoma ranged between one and 14. Following the OHTS study in 2002, the number of CCT and glaucoma articles has significantly increased and now ranges between 19 and 99 annually [6]. Many such studies have consolidated and expanded on previous findings, endorsing the notion that CCT plays a significant role in glaucoma management [2]. Indeed, the prognostic significance of CCT is now recognized not only in patients suspected of having glaucoma but also in monitoring the progression and severity of the disease in POAG patients. Analysis has shown that patients with more advanced glaucoma-related damage are more likely to have thin corneas [12]. The association between thin CCT and glaucomatous damage has been tentatively attributed to a link between a thinner cornea and a weaker optic nerve lamina cribrosa [13]. Whether merely as a factor affecting IOP measurements or an independent risk factor for glaucoma, accurate CCT measurements are paramount in optimizing patient care.

In clinical practice, various methods exist to measure CCT. Currently, ultrasound pachymetry (USP) is widely viewed as the gold standard due to its high degree of reproducibility, as well as its relative speed and ease of use [14]. USP requires contact with the corneal surface, and hence it utilizes echo spike techniques and the Doppler Effect to determine CCT [15]. More recently, non-invasive dedicated anterior-segment OCTs (AS-OCTs) have been developed. These instruments utilize low-coherence light to provide the user with high-resolution, cross-sectional images of the anterior segment [16]. The CASIA2 (Tomey Corporation, Nagoya, Japan) is a second-generation Fourier-domain AS-OCT system that allows global scans of the entire anterior segment [17]. Included in such scans is a measurement of CCT. Both USP and CASIA2 methods are available for use at the Royal Hallamshire Hospital (RHH) in Sheffield, UK. To ensure consistency and reliability in CCT measurements, it is imperative that the degree of agreement between these methods is assessed and recognized.

Several previous studies have compared various methods of measuring CCT [14,8-24], and many such studies have examined the degree of agreement between USP and the original model of CASIA (CASIA SS-1000) [22-24]. In this study, we investigate the degree of agreement between USP and the second-generation CASIA model, CASIA2. To the best of our knowledge, this is the first study to engage in a comparison of these two methodologies. Additionally, we discuss the possible reasons underpinning any observed differences.

## Materials and methods

The current study was undertaken at RHH, Sheffield. Patients suspected of having glaucoma who are referred from primary and secondary care attend the RHH glaucoma-screening service. These patients undergo Snellen chart visual acuity tests, IOP measurement, optic disc imaging (OCT and non-mydriatic color photography), visual field assessment, gonioscopy, and USP. All assessments are performed by trained and experienced clinicians. Patients with suspected narrow iridocorneal angles are also imaged using CASIA2. The standard operating procedure for iridocorneal angle scans using the CASIA2 automatically measures CCT. Thus, a proportion of glaucoma-screening patients undergo two separate CCT measurements (per eye), using USP and CASIA2 during the same appointment. This presents an opportunity to assess the degree of agreement between USP and CASIA2 in terms of CCT measurements in a clinical setting.

The measurements used in this study were collected retrospectively by accessing the records of glaucoma-screening appointments from January to March 2020, resulting in a dataset of 156 eyes. USP was performed using the DGH 55 handheld pachymeter (Pachmate, DGH Technology, Exton, PA). The pachymeter is calibrated weekly within the department. Before the measurement was performed, each eye was anesthetized using oxybuprocaine hydrochloride 0.4% eye drops. The patients rested their chin and forehead against a sterilized slit lamp headrest. While the patients fixated on a marker directly ahead, the pachymeter probe was placed as perpendicularly as possible to the center of the cornea. Twenty-five readings were obtained, and the mean value plus standard deviations (SD) were recorded.

CASIA2 measurements were performed within 15 minutes of USP measurements. Patients rested their chin and forehead on a sterilized headrest and were instructed to gaze toward a central fixation light. Images were acquired using the global AC analysis exam protocol and exported to the departmental image database. To obtain CCT measurements, the CCT option of the 2D analysis was selected. Before a CCT reading was recorded, the automated alignment of the posterior and anterior corneal surface markers was checked by the clinician to ensure the accuracy of the reading.

The CCT values measured by USP and CASIA2 of 156 eyes were analyzed and descriptive statistics were performed to calculate the mean, median, SD, and maximum and minimum CCT values for both measuring methods. To assess the linear relationship between the two methods, a Pearson correlation coefficient and a linear regression were performed. To test for a significant difference between the two methods, a student’s paired t-test was used. To analyze the relationship between the average CCT of the same eye [(USP CCT + CASIA2 CCT)/2] and the difference between the two measurements, a Pearson correlation and a linear regression were performed. Additionally, a Bland-Altman plot was constructed to visualize the limits of agreement between the two methods [25]. Finally, to assess the relationship between USP measurement standard deviations and the observed differences between the two methods, a Pearson correlation was performed. This aimed to establish whether any differences in CCT between the methods could be due to variations in the SD of USP measurements. All statistical analyses were performed using R version 3.6.3 [26]. Plots were created using ggplot2 [27].

## Results

In this study, the CCT values of 156 eyes from 78 patients were analyzed. The median age of the patients was 66 years (range: 20-86 years). The mean CCT value obtained from USP was 555.97 ± 40.81 µm. In comparison, the mean CCT value obtained by using CASIA2 was 535.99 ± 38.65 µm. The mean difference between the two methods, when measuring CCT of the same eye, was 19.98 ± 10.78 µm. Additionally, 88% of eyes differed by 10 µm or more between the devices.

**Figure 1:**
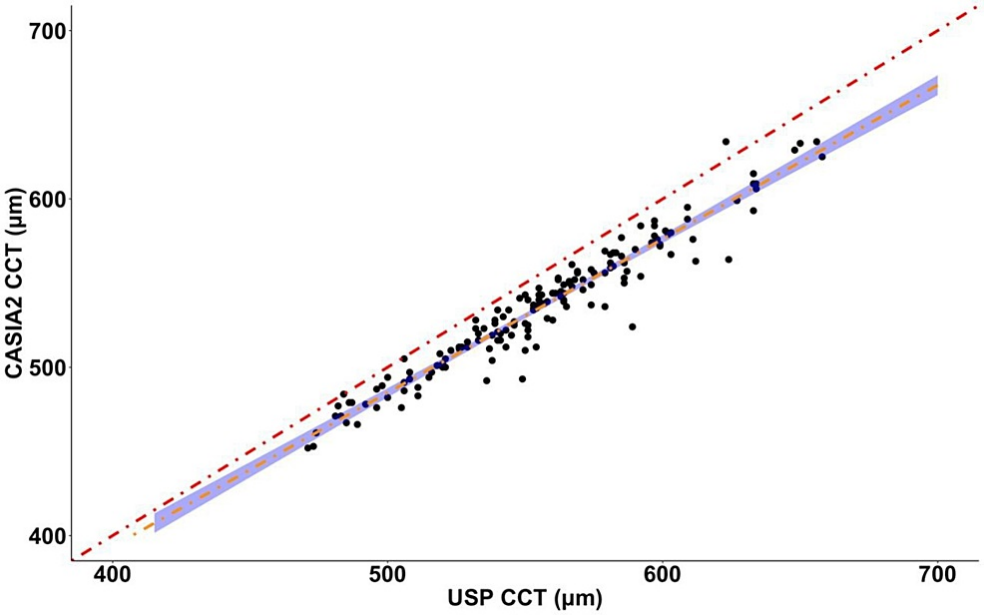
Relationship between USP CCT and CASIA2 CCT measures Each point represents an eye measured with both methods. Pearson correlation: r=0.97, t=45.41, p<2.2 x 10^-16^. The red line shows y=x (line of agreement). The orange line is the regression line (R^2^=0.93; p<2.2 x 10^-16^) with 95% confidence intervals USP: ultrasound pachymetry; CCT: central corneal thickness

**Figure 2:**
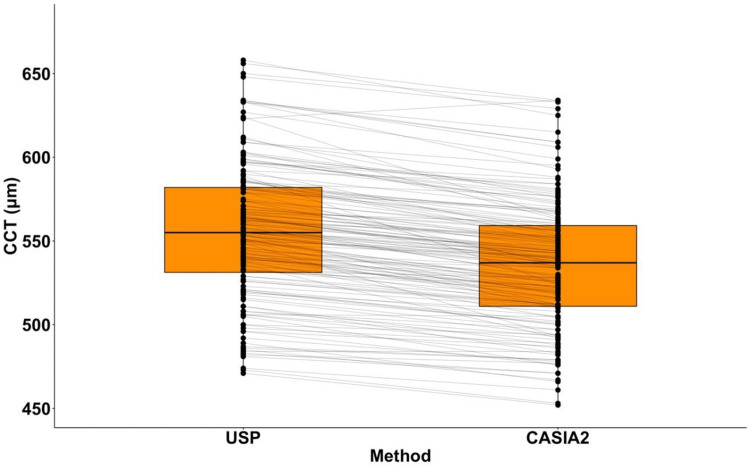
Distribution of CCT values for USP and CASIA2 Boxes represent the interquartile range with the median shown within. Horizontal lines join measurements of the same eye. The means are significantly different (paired student’s t-test: t=23.15, df=155, p<2.2 x 10^-16^) USP: ultrasound pachymetry; CCT: central corneal thickness

The CCT values obtained from USP were positively correlated with those measured using CASIA2 (Pearson correlation: r=0.97, t=45.41, p<2.2 x 10^-16^) (Figure 1). Despite the significant correlation, the lower CASIA2 values are demonstrated by the linear regression line (R^2^=0.93, t=45.41, p<2.2 x 10^-16^) falling below the y=x line (hypothetical line of total agreement between methods). The observed difference between the methods was found to be significant (paired student’s t-test: t=23.15, p<2.2 x 10^-16^) (Figure 2).

**Figure 3:**
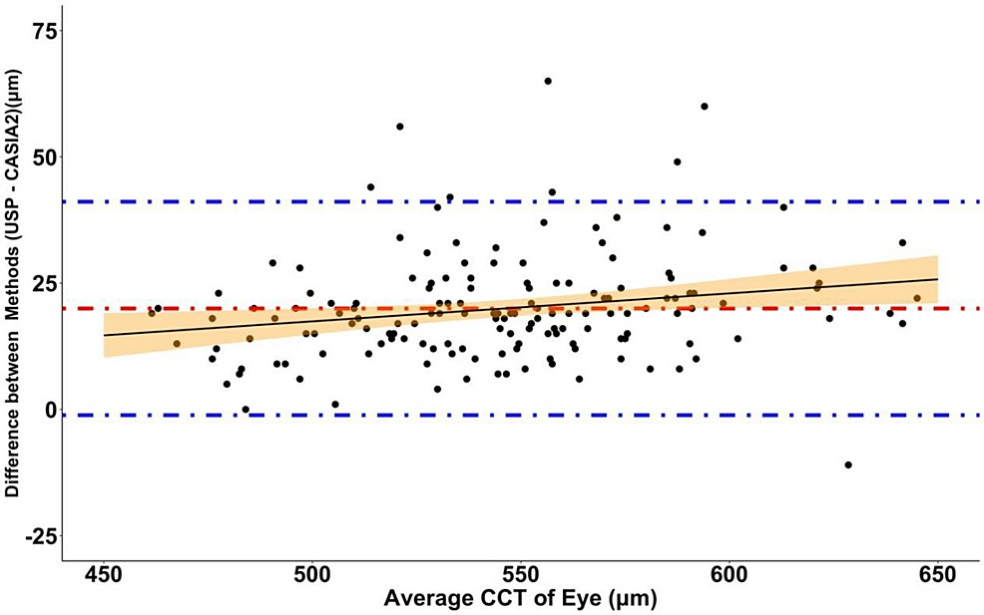
Bland-Altman analysis plot showing the difference between methods vs. average CCT of the same eye using the two measuring methods The red line indicates the mean difference of 19.98 μm. Blue lines indicate 95% limits of agreement (-1.15 μm to 41.11 μm). Pearson correlation: r=0.20, t=2.57, p=0.01. The black line indicates a linear regression line (R^2^=0.04, p=0.01) with 95% confidence intervals CCT: central corneal thickness

**Figure 4:**
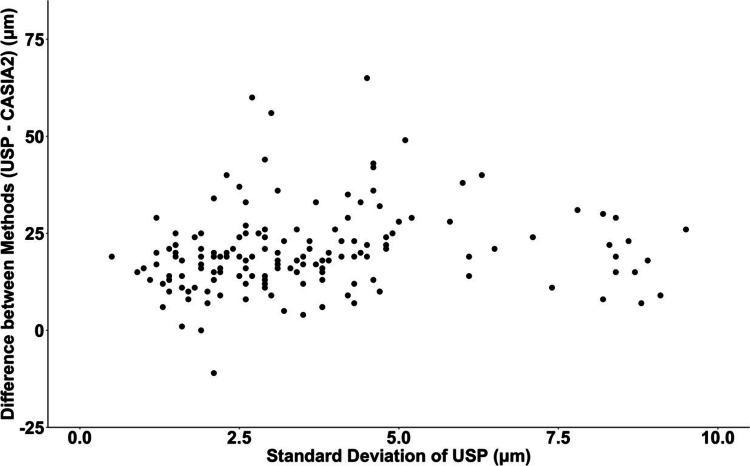
Relationship between the standard deviation of USP measurements and the difference between methods Pearson correlation: r=0.16, p=0.049 USP: ultrasound pachymetry

The limits of agreement between the two methods are demonstrated by Bland-Altman analysis (Figure 3). The 95% limits of agreement (mean ± 1.96 SD) range from -1.15 μm to 41.11 μm. That is, for 95% of eyes, the CCT value given by USP would be between -1.15 μm and 41.11 μm more than the values measured by CASIA2. The observed differences between the methods were significantly but weakly positively correlated with the average CCT [(USP CCT + CASIA2 CCT)/2] of the eye (Pearson correlation: r=0.2, t=2.57, p=0.01). Finally, there was a statistically significant but very weak correlation between the observed differences when comparing the methods and SD of USP measurements (Pearson correlation: r=0.16, t=1.99, p=0.049) (Figure 4).

## Discussion

Due to its effect on IOP measurements, it is of paramount importance to obtain CCT readings as part of a glaucoma assessment [5]. Although USP is the most widely utilized CCT measuring method, it is not without its disadvantages [15,18]. As a contact procedure, USP carries the risk of infection and corneal epithelial damage and can cause patient discomfort [18]. Additionally, accurate and repeatable USP measurements rely on the operator placing the probe as centrally and as perpendicular to the corneal surface as possible. The alignment and perpendicularity of the probe may significantly vary as they rely upon the operator’s judgment [18]. In recent years, various non-contact CCT measuring methods have been developed. The CASIA2 is an AS-OCT that provides the user with CCT measurements obtained optically. In this study, we investigate the degree of agreement between these two investigative methods.

The results of this study suggest a statistically significant difference between the CCT values obtained from USP and those from CASIA2. When measuring the same eye, USP consistently produced thicker CCT readings than CASIA2. The mean difference between CCT measurements of the same eye was 19.98 ± 10.78 μm. Such results broadly agree with previous studies that have assessed methods of measuring CCT. For example, many studies have found USP CCT readings to be significantly thicker than OCT-acquired CCT readings. USP has been found to measure the same CCT 14.4-49.4 μm thicker than time-domain OCT [20]. Similarly, USP has also been found to measure the same CCT thicker, at a value ranging from 7.9 μm to 19.7 μm, than Fourier-domain OCT [14,18-21]. Our results are also in agreement with those from comparisons between USP and the first-generation CASIA model (SS-1000). USP has been found to measure the same CCT thicker, between 9.3 μm and 16 μm than the CASIA SS-1000 [22-25]. The current study is possibly the first to demonstrate the same pattern when comparing the latest CASIA model (CASIA2) with USP.

**Figure 5:**
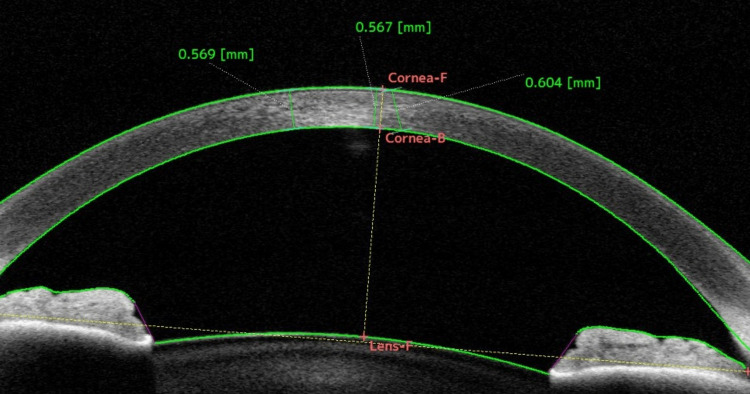
Screenshot of CASIA2 2D-analysis function screen Lines are drawn manually across the cornea to demonstrate thickness at different locations and differing degrees of perpendicularity. Line A is off-center but perpendicular to the corneal surface. Line B is centered and perpendicular to the corneal surface. Line C is centered but not perpendicular to the corneal surface

The differences observed between CCT values from USP and OCT devices may arise due to a variety of reasons. Firstly, the local anesthetic drops applied prior to USP may cause transient corneal edema. Studies have shown that the application of oxybuprocaine hydrochloride 2.5% drops can increase CCT by approximately 8 μm. However, CCT typically returns to its baseline value after 80 seconds [28]. This temporary increase in thickness may contribute to the difference observed in the current study as the time between USP and CASIA2 measurements was significantly more than 80 seconds. Secondly, USP CCT measurements are associated with a degree of uncertainty as the location of corneal posterior surface reflection may vary. The reflection point at the posterior surface may be located posterior to the endothelium (at the viscous film between the endothelium and anterior chamber) [29]. Additionally, a high standard deviation of USP measurements could lead to unreliable readings and potentially cause differences between this method and AS-OCT. However, the standard deviation of USP measurements had only a very weak correlation with the difference between USP and CASIA2 CCT values (Figure 4). That is, even USP measurements with low standard deviation values (ostensibly more accurate readings) differed significantly from CASIA2 measurements of the same eye. Finally, inaccurate USP probe placement may lead to artificially thicker CCT measurements [14,20]. To examine this hypothesis, the CASIA2 2D analysis function was used to draw measurement lines at three different corneal locations: (A) off-center and perpendicular, (B) central and perpendicular, and (C) central and non-perpendicular (Figure 5).

These measurements indicate that, even when centralized, USP measurements with non-perpendicular probe placement could produce much thicker CCT values than the actual CCT (C=604 μm compared with B=567 μm). The less perpendicular the probe placement, the longer the distance the sound waves must travel between the corneal anterior and posterior. This may also explain the positive, albeit weak, correlation between the difference and the average CCT (Figure 3). Probe placement slightly oblique to the corneal surface may lead to increasingly elongated measurements in eyes with thicker CCT. As perpendicularity is reliant on clinician judgment and patient compliance, it is not guaranteed and can vary significantly. This differs from CASIA2 measurements, as centralization and perpendicularity are standardized based on a line bisecting a second line joining the nasal and temporal angle recesses, thus accounting for wayward patient fixation or clinician misjudgment. The authors hypothesize that the differences in CCT measurements at different levels of perpendicularity to the central corneal surface may drive the difference in USP and CASIA2 measurements observed in this study.

Regardless of the reasons underpinning the observed data, it is important to question whether such differences would be clinically significant. Correction factors are commonly used in glaucoma clinics to “correct” IOPs based on an eye’s CCT. Such factors generally use a linear scale correction factor, which suggests an increase or decrease in IOP by a given amount according to the CCT [30]. For example, a commonly used correction factor is provided with the Pachmate pachymeter. Based on studies where anterior chambers were cannulated to measure true intracameral IOP [8], this correction factor suggests a 1-mmHg IOP adjustment for every 10-μm deviation from 545-μm CCT. According to this scale, the 19.98-μm average difference between USP and CASIA2 measurements found in this study would mean the corrected IOP of an eye may differ by 2 mmHg (19.98 μm ~2 x 10μm) based on the measuring method used. It should be stated that the accuracy of such correction scales has been questioned [30]. Most assume a linear relationship between CCT and its effect on IOP measurements, but this has been shown to be inadequate due to corneal biomechanical complexities. This has been shown in cases with lower IOPs, where CCT makes less of an impact [30]. Nonetheless, the observed difference between USP and CASIA2 in this study may still be clinically relevant.

The OHTS revealed that a thin CCT is a powerful risk factor for the development of POAG. The OHTS suggests that a mere 33-μm difference (588 to 555 μm) in CCT can increase the chance of developing POAG by three-fold [12]. The Bland-Altman analysis (Figure 3) demonstrated that the differences between USP and CASIA2 for 95% of eyes measured may range from -1.15 μm to 41.12 μm. Therefore, an eye measured with USP may not seem at risk due to a thin CCT, but the same eye measured with CASIA2 could have a CCT up to 41.12 μm thinner, and thus the patient could be judged to be at a significantly increased risk of developing POAG. However, it must be stated that, since 2017, National Institute for Health and Care Excellence (NICE) no longer recommends the use of CCT in glaucoma treatment algorithms. However, the same guidelines do still list CCT as an important risk factor for the development of glaucoma and must therefore always be considered [5].

This study has a few limitations. Firstly, measurement methods could not be fully standardized. As measurements were obtained during hospital clinic visits, the measurements were performed by multiple clinicians. Although departmental standard operating procedures exist, interoperator technique variability is inevitable. Secondly, this study used both eyes from individual patients. Treating each eye as an independent value carries the risk of results reflecting pseudo-replication. However, all statistical tests were performed using the CCTs of the right eye (one measurement from each patient). Although this reduced the sample size, the results were very similar to those obtained using the larger sample size. That is, when examining the CCTs of only the right eyes, there was still a significant difference between USP and CASIA2 (a difference of 19.38 μm; student’s paired t-test, t=17.01, p<2.2e-16).

## Conclusions

This study compared the CCT values obtained from USP and CASIA2 and found a significant difference between the two methods. When measuring the same eye, USP measured the CCT at an average of 19.98 μm thicker than CASIA2. The observed differences in range measurements of CCT, -1.15 μm to 41.11 μm, between USP and CASIA2 were statistically significant. Consequently, these observed differences in measurements can have a significant effect on clinical assessment and judgment. The variations between the measured CCTs will directly affect risk stratification for patients at risk of developing glaucoma. In addition, using the two measurement devices interchangeably for the assessments and follow-ups of the same patients may also have an erroneous bearing on prognosis. Therefore, we recommend that the two methods not be used interchangeably, for the same patient at the very least, and clinicians should take cognizance of the significant differences observed in this study. Future studies will need to assess any differences between USP and CASIA2 CCT measurements when controlling for pre-assessment application of oxybuprocaine hydrochloride 2.5% drops (which may result in transient increases in CCT).
